# Energy expenditure and weight-related behaviors in youth with Down syndrome: a protocol

**DOI:** 10.3389/fped.2023.1151797

**Published:** 2023-07-20

**Authors:** Michele Polfuss, Linda G. Bandini, Michele N. Ravelli, Zijian Huang, Andrea Moosreiner, Dale A. Schoeller, Chiang-Ching Huang, Dan Ding, Cristen Berry, Emma Marston, Azeem Hussain, Timothy C. Shriver, Kathleen J. Sawin

**Affiliations:** ^1^ College of Nursing, University of Wisconsin - Milwaukee, Milwaukee, WI, United States; ^2^ Department of Nursing Research and Evidence-Based Practice, Children’s Wisconsin, Milwaukee, WI, United States; ^3^ Eunice Kennedy Shriver Center, University of Massachusetts Chan Medical School, Worcester, MA, United States; ^4^ Isotope Ratio Mass Spectrometry Laboratory, Biotechnology Center, University of Wisconsin, Madison, WI, United States; ^5^ Department of Rehabilitation Science and Technology, University of Pittsburgh, Pittsburgh, PA, United States; ^6^ Clinical and Translational Science Institute of Southeast Wisconsin, Medical College of Wisconsin, Milwaukee, WI, United States; ^7^ Zilber School of Public Health, University of Wisconsin – Milwaukee, Milwaukee, WI, United States; ^8^ Pediatric Translational Research Unit, Children’s Wisconsin, Milwaukee, WI, United States

**Keywords:** Down syndrome, trisomy 21 (Down syndrome), nutrition, physical activity, energy expenditure, obesity, doubly labeled water (DLW), wearable devices

## Abstract

**Background:**

The consequences of obesity are ominous, yet healthcare professionals are not adequately preventing or treating obesity in youth with Down syndrome (DS). Total daily energy expenditure (TDEE) is the energy expended in 24 h through physical activity and life-sustaining physiologic processes. An individual's TDEE is essential for determining the daily caloric intake needed to maintain or change body weight. Successful prevention and treatment of obesity in youth with DS is severely compromised by the lack of data on TDEE and information on weight-related behaviors for this high-risk population. This manuscript describes the protocol for the federally funded study that is in process to determine daily energy expenditure in a large cohort of children with DS.

**Methods:**

This observational cross-sectional study will include a national sample of 230 youth with DS, stratified by age (5–11 and 12–18 years of age) and sex. Doubly Labeled Water analysis will provide the criterion body fat%, fat-free mass, and TDEE. To increase accessibility and decrease the burden on participants, the entire study, including obtaining consent and data collection, is conducted virtually within the participant's home environment on weekdays and weekends. The study team supervises all data collection via a video conferencing platform, e.g., Zoom. This study will (1) examine and determine average TDEE based on age and sex, (2) develop a prediction equation based on measured TDEE to predict energy requirements with a best-fit model based on fat-free mass, sex, age, and height and/or weight, and (3) use 24-hour dietary recalls, a nutrition and physical activity screener, wearable devices, and sleep questionnaire to describe the patterns and quality of dietary intake, sleep, and physical activity status in youth with DS.

**Discussion:**

The lack of accurate information on energy expenditure and weight-related behaviors in youth with DS significantly impedes the successful prevention and treatment of obesity for this vulnerable population. The findings of this study will provide a further understanding of weight-related behaviors as obesity risk factors, currently not well understood for this population. This study will advance the science of weight management in individuals with disabilities and shift clinical practice paradigms.

## Introduction

The consequences of obesity are ominous, yet we are not adequately preventing or treating obesity in youth with Down syndrome (DS), who have a dramatically higher obesity prevalence (reported as high as 62.5%) (1) compared to their typically developing peers (18.5%) (2). Obesity is associated with life-long medical, economic, and psychological burdens which worsen with earlier age of onset (3–5). In children with disabilities, obesity limits independence, decreases the ability to self-manage health, increases the risk of social isolation, and is a barrier to caregivers' abilities to provide care (6, 7). In DS, obesity is linked to adverse health outcomes such as obstructive sleep apnea, dyslipidemia, hyperinsulinemia, impaired cardiorespiratory fitness, and orthopedic complications (1, 6–14). With dramatic increases in life expectancy for individuals with DS, it is imperative to ensure that they enter their adult years with optimal health (15). To address obesity in youth with disabilities, the National Institute of Child Health and Human Development (NICHD) expert panel's research agenda prioritized (1) addressing the need for accurate data on energy expenditure and (2) identifying and understanding weight-related behaviors as obesity determinants to inform potential interventions (16).

Obesity in youth often continues into adulthood impacting morbidity and mortality (17, 18). In the simplest terms, obesity is an outcome of an imbalance of excess energy intake as compared to energy expended (physical activity and physiologic processes) described for a 24-hour period as total daily energy expenditure (TDEE) (19). Characteristics inherent or related to DS (e.g., hypotonia, decreased fat-free mass, hypothyroidism, leptin resistance, less participation in physical activity) are associated with decreased TDEE (1, 12, 16, 20–24). These factors contribute to a lower level of energy expenditure resulting in a reduced caloric need and consequently increased risk of inadvertent overfeeding and subsequent weight gain (25). In addition, individuals and parents often overestimate the amount of energy expended through physical and sedentary activity further adding to unintentional excess intake (26–30). Growth retardation, decreased height velocity and muscle hypoplasia can further exacerbate the high percentage of body fat and can be accentuated with the youth's advancing age (1).

An individual's TDEE is essential for determining the energy intake required to maintain or change body weight (31) and is the foundation of anticipatory guidance provided by healthcare professionals to optimize growth and weight management. Specifically, the very limited data on the energy needs of youth with DS does not allow accurate recommendations for dietary intake (32). Successful prevention and treatment of obesity in youth with DS is severely compromised by the lack of data on TDEE and an understanding of weight-related behaviors.

Behaviors that contribute to obesity often begin in childhood or adolescent years (33). The onset of obesity in youth often continues into adulthood (17, 18, 34). Limitations in the current literature include a distinct void of inclusion of individuals with DS in weight-related research (23, 35). When individuals with disabilities are the focus of the study, limited studies have used “state-of-the-art measurement techniques” [i.e., doubly labeled water (DLW)] (24). While preliminary studies providing support for youth with developmental disabilities having poor quality diets, increased screen time, and needing less caloric intake per day are present, they are limited and recommend further study in larger samples (1, 16, 20, 23, 24, 36–38). Focusing on the prevention and treatment of obesity in youth with DS is a national priority (16, 39).

This study focuses on youth with DS and was a competitively reviewed supplement to our currently funded Body Composition and Energy Expenditure in Youth with Spina Bifida (R01HD096085). While the protocol is similar to the initial R01 aim addressing TDEE in children with spina bifida (SB), this protocol differs in the population of interest and specifics of the design, specifically the setting, recruitment, methods of data collection, and addition of DS-focused measures.

This protocol addresses gaps and weaknesses of prior research in this cohort as it pertains to establishing caloric need. This study will systematically investigate TDEE and develop an algorithm for use in youth with DS as stratified by age and sex to predict energy requirements. As a result, recommendations of daily caloric intake will be established. The second outcome will be information related to obesity determinants (i.e., dietary intake, sleep, and activity) in youth with DS.

The study aims are:

Aim 1. Using DLW, measure TDEE and develop a prediction equation for the energy requirements of youth with DS. We propose to: (a) Examine and describe average TDEE stratified by age and sex and (b) Develop a prediction equation based on actual TDEE to predict energy requirements with a best-fit model based on fat-free mass, sex, age, and height and/or weight.
Aim 2. Using 24-hour dietary recalls, a nutrition and physical activity screener, accelerometers, activity trackers, and a sleep questionnaire, describe the patterns and quality of dietary intake and sleep, and duration and frequency of activity (physical and sedentary) in youth with DS.

## Methods

### Design and participants

This observational, cross-sectional study will include a national sample of 230 youth with DS, stratified by age group (5–11 and 12–18 years of age) and biological sex (Table 1). The study protocol is approved by the Western Copernicus Group (WCG) Institutional Review Board (IRB) (#20214186) and acknowledged by the local IRB of the Principal Investigator (PI).

**Table 1:** Sample stratification.

Sample stratification (*n*)		
*N* = 230	Male	Female
5–11 years	57	57
12–18 years	58	58

### Pilot study

This application was supported by our pilot study (P20NR015339 and UL1TR000055) that confirmed the feasibility of measurement of energy expenditure with DLW in youth with DS, SB, and without disabilities. In this small sample, TDEE was significantly lower in youth with disabilities. When matched for fat-free mass, TDEE in youth with DS averaged 500 fewer calories per day to balance their caloric intake compared to youth without disabilities (32).

#### Setting

The proposed study is conducted virtually with consent, data collection, and testing occurring within the participant's home environment via a HIPAA-compliant video conferencing platform. The decision to conduct the study virtually was done to minimize the study burden and to increase accessibility for participants to join from anywhere in the lower 48 United States. Hawaii and Alaska were excluded due to shipping costs.

#### Coordinating sites

This study is coordinated through the two agencies where the PI holds a joint appointment as the Joint Research Chair in the Nursing of Children, Children's Wisconsin, a free-standing Children's Hospital, and the University of Wisconsin—Milwaukee College of Nursing, both located in Milwaukee Wisconsin. The coordination center for assembling study kits, shipping, receiving, and sterilizing supplies, and processing and storing samples is through the Pediatric Translational Research Unit (PTRU) located within Children's Wisconsin. Accelerometer and activity tracker analysis is completed at the Department of Rehabilitation Science and Technology at the University of Pittsburgh and DLW is supplied and analyzed through the Isotope Ratio Mass Spectrometry (IRMS) lab of the University of Wisconsin—Madison.

### Recruitment, screening, and consent

Primary recruitment strategies include the use of the National Institutes of Health (NIH) DS-Connect®, a national registry that connects individuals with DS and their families to research and healthcare providers. Once a study is reviewed and approved by the registry, the study description is shared with registry participants who meet inclusion and exclusion criteria (Table 2). In addition, the study is shared with family-focused DS organizations and their associated social media sites, specifically targeting organizations that have a diverse focus, and the use of snowball recruitment is incorporated. Through each of these recruitment methods, the interest in participating in the study is participant-driven to allow them to make an informed decision to participate.

**Table 2:** Inclusion and exclusion criteria and rationale.

Inclusion criteria
Youth between the ages of 5–18 years old diagnosed with Down syndrome • Down syndrome is the focus of the study and ages 5–18 include youth during active growth and development when habits are being formed and there is a higher likelihood of being toilet trained and able to follow instructions with a parent or legally authorized representative (LAR) assistance.
Individuals who are English or Spanish speaking • Language study team has fluency in or access to a translator.
Access to Wi-Fi and basic capabilities with technology • Required for study completion.
Reside in the continental or lower 48 states of the United States • Hawaii and Alaska are excluded due to shipping costs associated with their geographical location.
Enrollment assures an equal split between males and females (115 in each group) • To analyze our data based on sex, as differences in energy expenditure are expected.
Live with a parent or LAR who is able to read/write/speak English or Spanish • Parent or LAR status and ability to read/write/speak English or Spanish are necessary for understanding the study materials and completion of the study protocol.
Exclusion criteria
Traveling >200 miles the week before or during the study protocol • Traveling >200 miles outside of the participant's primary residence during the protocol can impact the Doubly Labeled Water (DLW) results due to a shift in the natural abundances of the added isotopes (2H and 18O) in drinking water that varies per geographical region (40, 41).
Uses supplemental oxygen • The use of supplemental oxygen impacts the accuracy of one or more study measures.
Have medical restriction(s) to a 6-h fast • A minimum of a 6-h fast is recommended for the DLW test. For this reason, morning appointments will be recommended, and an approved snack will be offered to the participant early in the study protocol after baseline urine samples are completed.
Underwent a blood transfusion or IV infusion of >500 ml of IV fluids the week prior to the test date or are expecting to have during the study protocol • A blood or IV infusion >500 ml will negatively impact the accuracy of one or more study measures.
Are pregnant • Being pregnant will negatively impact the accuracy of one or more study measures.
Uses a g-tube for nutritional intake • The use of a g-tube for nutrition will provide atypical results for the dietary intake assessment.
Unable to stand independently or safely • The inability to stand independently or safely will interfere with the assessment of a standing height and performance of aspects of the study protocol.
Has an active viral or bacterial illness on the first day of testing • Having an active illness during data collection can impact the participant's hydration status, nutritional intake, and physical activity level.

Potential participants who are interested contact the study team through email or phone. A member of the study team meets with the potential participant via HIPAA-compliant video conferencing platform to review the study details, answer questions, screen for eligibility, and share additional study materials with them (e.g., a pictorial orientation booklet that provides a broad overview of the study protocol in an easy-to-read, child-friendly format). This allows the families to further consider and discuss as a family unit while making an informed decision to participate. If eligibility is confirmed and interest in the study remains, the team member performs the consenting/assenting (hereafter, consenting) process.

Consenting occurs via HIPAA-compliant video conferencing platform. This assists in ensuring the family's capabilities of using the virtual platform, and having sufficient Wi-Fi, and allows the consenting process to occur while seeing faces and/or body language to assist in confirmation of understanding and initiating the researcher-participant relationship that is helpful in the successful completion of the study. The study meets the requirements for a waiver of documentation of written consent under 45 CFR 46.117(c)(21)(ii). The consent forms are reviewed with the parent or legally authorized representative (LAR) by a Collaborative Institutional Training Initiative (CITI)-trained study team member. Due to the varying degrees of cognitive delays that may limit the ability to assent the IRB approved a waiver of assent. However, the study team member works with the parent/LAR to assess if the child is able to give verbal assent based on their maturity, psychological state, and cognitive ability on a case-by-case basis. While waiver of written documentation is present, all families are provided with a copy of the consent form for their records. All consents are professionally translated into Spanish. A data collector fluent in Spanish is employed for any families that primarily speak Spanish.

Upon informed consent completion, the study visit is scheduled. To accommodate families' schedules, weekday and weekend visits are available, preferably close to when the child wakes up due to early fasting requirements. Drinking up to 8 ounces of water and using a spoonful of yogurt or food for medication intake is acceptable if needed.

### Study procedures

**Figure 1:**
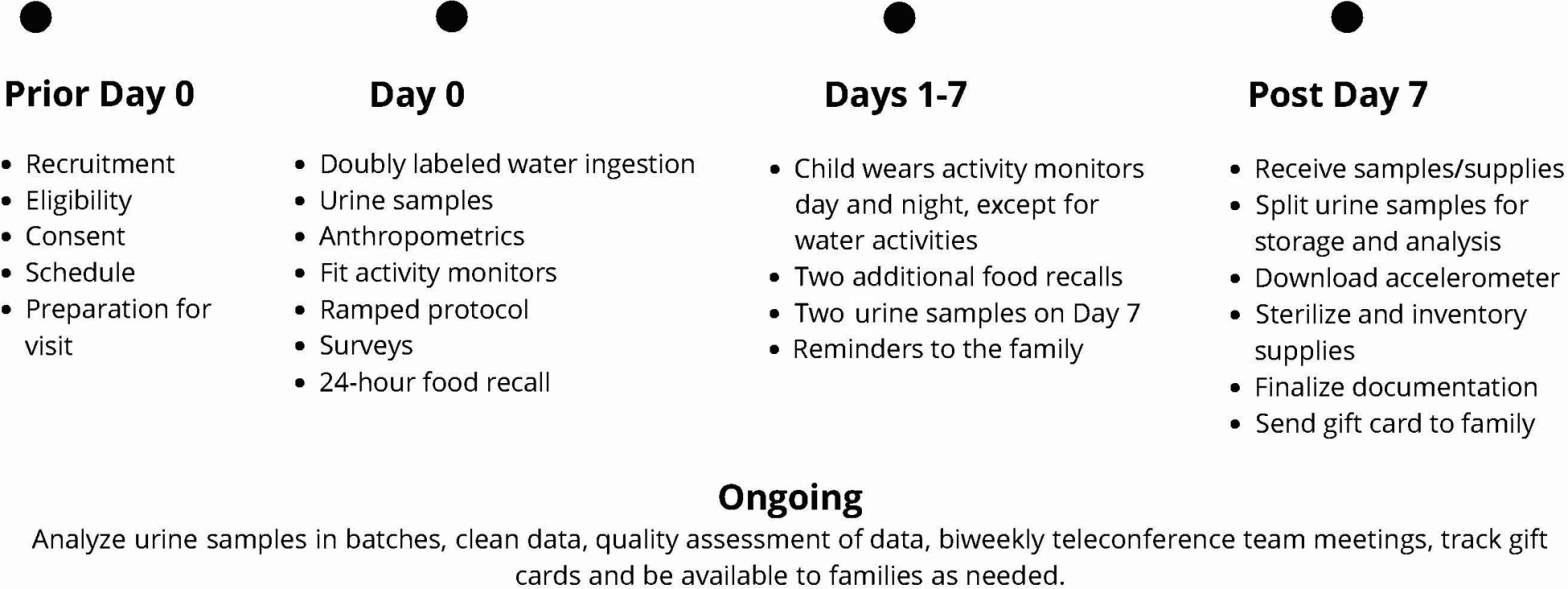
Timeline of study protocol.

The study includes an 8-day protocol that is conducted virtually within the family's home with a trained study team member working with the family via a HIPAA-compliant video conferencing platform, phone (call and text), and email communication. See Table 3 for a detailed study protocol and Figure 1 for a timeline of the study protocol. Once consenting has been completed, the parent/LAR is asked to provide an estimate of the child's weight (to dose DLW), share their home address to send supplies, and schedule their visit (Day 0). This initial data collection visit is estimated to last 4.5 h but it is recommended that 5 h be scheduled in case of unforeseen issues. Prior to the scheduled visit date, two boxes are shipped to the family home that includes the supplies needed to execute the study protocol. All materials are clearly marked, color-coded, and have an easy-to-read family-friendly procedure manual with them.

**Table 3:** Study protocol overview.

Prior to Day 0	
Recruitment and consent	Recruit participants and consent/assent via virtual platform, e.g., Zoom. This will also confirm participants’ Wi-Fi availability and ability to use computerized technology and address any concerns prior to data collection.
Preparation	Schedule study visit. Mail study supplies to the family. The week of the visit, meet with the family to confirm Day 0 virtual visit date and time, fasting requirements, no active illness, review supplies, and baseline urine collection.
Day 0 (5-h virtual visit)	
DLW and urine collection • Obtain body weight. • Obtain pre-DLW baseline urine sample—timed and stored. This may be done with the first morning urine upon the child waking or during the start of the visit. • Provide DLW based on body weight followed by 50 ml drinking water to ensure DLW dose is consumed. • Obtain post-DLW consumption urine samples at hour 1 (discard), hour 3 (time and store), and hour 4 (time and store) after DLW is consumed. Anthropometric measures (between urine collections) • Standing height (with portable stadiometer). • Waist, hip, and neck circumference with a flexible tape measure. • Ramped protocol of perceived exertion while wearing an accelerometer. Snack (between hour 1 urine and hour 3 urine) • After hour 1 urine and before hour 3 urine sample, provide snack option (sent in study kit based on participant preference and caloric limitation). Surveys (between urine collections) • Parents/LAR to complete all instruments with child assistance as needed. • All participants will complete Demographics, Block Food Screener, Child Habits Sleep Questionnaire, Autism Rating Scale, PEDI-CAT, PROMIS Peer Relations, Tanner stage, and a 24-hour dietary recall. • Youth ages 8 and above: Block Kids Physical Activity Screener. Prior to the end of the appointment • Confirm correct labels and review storing the urine samples collected in the refrigerator. • Review the sleep/monitor wear log to be completed for the 7 days. • Instruct on continuously wearing the accelerometer and activity tracker for the 7-day study protocol except for water activities. • Schedule the two additional 24-hour dietary recalls being performed over the 7-day study protocol. After the end of the appointment • Input study information in the REDCap database.	
Days 1–7	
• Complete the two additional virtual visit 24-hour dietary recalls for a total of 2 weekdays and 1 weekend day per dates chosen by participant/parent/LAR. • Review and remind the family of the day 7 process of collecting two urine samples (1 h apart). • Instruct families to ship back urine samples and wearable devices on Monday, Tuesday, or Wednesday. • Provide reminders and periodic check-ins via phone and/or text and be available for family questions as needed.	
Post receipt of urine samples and supplies	
• Upon receipt of participant urine samples (Day 0 baseline, hour 3, hour 4, and day 7 hour 1 and hour 2), split all samples into two 5 ml cryotubes and freeze at −20°C. (One stays at PTRU for quality assurance, and one is mailed to IRMS Lab in Madison, Wisconsin in batches for analysis.). • Download accelerometer data, reset activity tracker and charge the devices for the next participant. • Sterilize and inventory all supplies. • Finalize participant information in the REDCap database • Email or mail gift cards to the family.	
Ongoing	
• Mail frozen urine samples (from each participant) to IRMS lab at UW-Madison. • Store the backup frozen samples (5 ml tube) within the pediatric research unit at −20°C. • Participate in biweekly and as-needed teleconference team meetings. • Clean and perform quality checks of the REDCap database. • Track gift cards.	

Box 1 includes supplies for measurement and assessment (portable scale for body weight, portable stadiometer, flexible tape measure, accelerometer, activity tracker, iPad for questionnaires, urine collection supplies (e.g., urine hats, urine cups, double bag, absorbent materials, preprinted labels, and parafilm), measuring cups, calorie-controlled snack, and paperwork. Box 2 is insulated and includes the DLW dose, shipping materials for returning urine samples and items used past Day 0 (accelerometer, activity tracker, ice packs). The week before the study visit, the family meets with a study team member to review fasting requirements, study kit materials, and the plan for the study visit including the collection of a baseline urine sample using the child's first-morning urine and refrigeration of DLW for taste purposes. In addition, written instructions, and materials for collecting urine samples as well as gloves and storage materials are provided. This allows the family to collect the baseline urine sample upon the child's waking but before the start of the virtual meeting depending on the start time of the visit.

On Day 0, a study team member connects via a video conferencing platform with the participant and a minimum of one parent/LAR. The child's fasting status and lack of any active illness are confirmed. If the family has not already collected a baseline urine sample, they are asked to collect this sample with the team member's guidance at the start of the visit. If they did collect the sample, confirmation of proper collection and timing of the sample is obtained. The parent/LAR is asked to obtain the child's body weight with excess clothing removed on the SECA 813 portable scale provided. The research assistant uses this weight to confirm that the dose of DLW is appropriate for the child's weight. If the DLW is spilled, the protocol would be stopped, the visit would be rescheduled with a new dose of DLW shipped and the baseline urine will be stored and analyzed. If the wrong dose was given and it is smaller than planned, the center staff calls the IRMS lab to discuss options.

The child drinks the DLW from the provided bottle and straw as spillage reduces accuracy. After the child drinks the DLW from the bottle that it was sent in, the parent/LAR is asked to add 50 ml of tap or bottled water to the now empty bottle with a provided measuring cup and a marked line identifying 50 ml on the DLW bottle. The child drinks the added water with the same straw used for the DLW. This ensures that all DLW is consumed. Three additional urine samples beyond the baseline sample are obtained during the Day 0 visit. At 1 h after the child drinks the DLW, a second urine sample is obtained and discarded to flush the bladder. Two urine samples are collected and saved at 3- and 4 h post DLW consumption. These two samples are labeled with the child's coded identification number, date, and time and stored in the refrigerator. An example would be if the DLW was consumed at 08:15 a.m., the Hour 1 urine sample is obtained at 09:15 a.m. (discarded), the Hour 3 urine at 11:15 a.m., and the Hour 4 urine at 12:15 p.m. (both labeled and refrigerated). If the child is unable to void, the child is asked to try again in 15 min. If still unable to void, 60 ml of drinking water is provided, and the child is asked to try again in 15 min. This continues until the child is able to void. If a urine sample is delayed, the timing of the next urine collection is adjusted based on the actual time of the urine sample. In between the urine sample collection, ample time is available to complete survey instruments and other study measures and to provide the family with downtime to be off camera as needed.

Between the timed urine sample collection, families are guided on the completion of additional measurements including a standing height with the SECA 213 portable stadiometer. A flexible tape measure is used to obtain waist, hip, and neck circumferences. These measures are collected under the guidance of study staff to confirm landmarks and fidelity of the measures. All circumferences are measured three times and the average of the measures is calculated. If an extreme outlier (>3 cm) is obtained, the parent/LAR is asked to obtain an additional measure. The study team member documents all measures in the REDCap database. A study-provided calorie-controlled snack is included in the study box to be available after Hour 1 urine but before Hour 3 urine per DLW protocol. The snack is discussed during consenting to confirm no allergies and preferences of the child. If a family prefers their own snack, confirmation from the study team is needed to ensure that the snack is 250 kcal or less. During this snack time, the child is also offered 8 ounces of water. This supports the collection of the Hour 3 and Hour 4 urine samples. The snack (kcal) and drink (ounces) are documented in the amount and time consumed.

In addition to anthropometric measurements, the family is asked to complete questionnaires and is guided to set up data collection devices and place them on their child. All questionnaires, except the Block Nutrition Screener and Block Physical Activity Screener are provided via REDCap on the study iPad that is provided. The study team member, who is trained in conducting 24-hour dietary recalls, works with the parent/LAR and child if able to assist, to document the child's previous day's intake using a multiple-pass approach. Measuring cups, spoons, and visual aids are included in the supplies for the family to assist in quantification and portion sizes. The final item is conducting a ramped protocol to assess physical activity. This includes the child wearing the study-provided preprogrammed accelerometer (ActiGraph GT3X-BT, ActiGraph LLC, Pensacola, FL, USA) on their waist and the activity tracker (Fitbit Inspire 2, Fitbit, Inc, San Francisco, CA, USA) on their non-dominant wrist. With guidance from the study team, the parent fits each device to their child, activation is confirmed, and the device is time synced. The activity tracker is configured to minimize or remove notifications for the child/family to decrease the risk of altering the child's health habits for the week. The team members guide the child with the parent/LAR's assistance through a ramped protocol divided into five intervals that include the child moving at light, moderate and vigorous activity levels, with perceived exertion reported at each level. The five intervals include (1) obtaining a resting heart rate (3 min), (2) standing, and wiping surfaces for arm movement (3 min), (3) walking at a normal pace (3 min), (4) walking at a fast pace (6 min), and (5) dancing in place (2 min). Between intervals, the child's heart rate must return to baseline before doing the next activity. The child and parent/LAR discuss and provide the child's perceived exertion rate for that interval based on a pictorial perceived exertion chart (42), This allows the study team to individualize the analysis of the activity tracker and accelerometer raw data for the study protocol.

After the Hour 4 urine collection, it is expected that Day 0 study requirements are completed. The study team uses the end of the visit to review the remainder of the protocol including the continued wearing of the accelerometer on the child's waist and the activity tracker on their non-dominant wrist during wake and sleep times but removed during water activities (e.g., bathing, swimming). The benefit of the activity tracker worn with the accelerometer is the continuous recording of heart rate and physical activity information from both research-grade and commercially available wearable devices. The family is provided with a daily wear/sleep log sheet and asked to document when the child goes to bed, wakes up, and is not wearing the accelerometer or activity tracker over the following week. Guidance is also provided to assist the family in repackaging equipment and supplies. Appointment times for two additional 24-hour dietary recalls are set up based on the family's availability to include a total of 2 weekdays and 1 weekend day. The family is asked to continue with their normal daily routine during the protocol. The final action item of the study is the collection of two remaining urine samples a minimum of 1 h apart on Day 7, preferably close to the time of day that Day 0 samples are collected. The study team reviews this during the final food recall and is available to assist the family if needed.

After the study protocol is complete and the final two urine collections are obtained, the parent/LAR is guided to ship the boxes back using a prepaid shipping label. Specific instructions to schedule optional home pick up of the boxes are included to add convenience for the family. The refrigerated urine samples are shipped back using the insulated storage box and frozen gel bags that were provided. The prepaid shipping label for the insulated box that includes urine samples is for overnight shipment and the family is instructed to only ship this box on Monday, Tuesday, or Wednesday of each week to ensure that personnel will be available to accept the shipment. The other supplies in the second box use a prepaid shipping label for ground transportation. Upon receipt of the urine samples and supplies, the family is provided an e-gift card for $250.00 through email. If the family is uncomfortable with the electronic gift card, a physical gift card can be mailed via the United States Postal Service, but a signature will be needed to confirm receipt.

The two boxes are shipped back to the coordination center (i.e., PTRU Children's Wisconsin) where the urine samples are processed, documented, and split into two. One sample is frozen and remains at the PTRU for quality control purposes. The second sample is mailed in batches to the UW-Madison IRMS Lab for analysis. All samples are de-identified and labeled with the time and date of collection and participant study identification number. The remaining equipment and supplies are inventoried, sterilized, and confirmed to be in good working condition.

### Measures

#### Anthropometric measures

To provide contextual support for body composition and risk factors for obesity-related comorbidities the participant's weight, height, waist, hip, and neck circumference are obtained. Circumference measures are repeated three times with the average used for analysis. All measures are obtained by the parent/LAR with direction and guidance provided by the study team during the virtual session. Pictorial directions for measurements are also provided in a family-friendly procedure manual. See Table 4 for detailed measures and procedures.

**Table 4:** Anthropometric measures (weight, height, and body composition/circumferences).

Measures	Procedural operations
Weight (measured to the nearest 0.1 kg)	
Body weight	Participants remove shoes, extra clothing (jackets, heavy clothing, jewelry), and/or assistive devices and are weighed on a SECA 813 portable scale with an extra wide, non-skid platform.
Height (measured to the nearest 0.1 cm)	
Standing height	Participant removes shoes and/or assistive devices and stands erect with back and heels against a portable SECA 213 stadiometer. The Stadiometer arm is brought to the top of the child's head.
Body composition/circumferences (measured to the nearest 0.1 cm, unless otherwise indicated)	
Waist circumference	Measured with a flexible tape measure at two different landmarks: the umbilicus and immediately above the right iliac crest at the mid-axillary line.
Neck circumference	Measured with a flexible tape measure placed lightly around the neck at the height where a shirt collar would rest. Reminder to not crane the neck or move skin downward.
Hip circumference	Measured with a flexible tape measure at the widest part of the hips.
Doubly labeled water (DLW)	On Day 0, verify a minimum of 6 h of fasting/light fluid ingestion. Approximately 1–3 ounces of DLW (dosed on body weight) is consumed through a straw. After drinking the DLW, 50 ml of drinking water is added to the empty cup and consumed by the child. Urine specimens are collected at baseline (before DLW), hour 1-post-DLW (discarded), 3 (kept), and 4 (kept) and refrigerated. Day 7 parent/LAR collects two additional urine specimens 1 h apart with provided supplies and refrigerates until shipped back to the study team.

#### DLW variables and calculations

DLW is a valid and reliable tool that provides a measure of body fat and TDEE (43). DLW uses drinking water mixed with two stable isotopes, deuterium (^2^H) and oxygen-18 (^18^O) which act as tracers when ingested. DLW is dosed based on the individual's body weight. The analysis is performed on the individual's body fluids (urine) by isotope ratio mass spectrometry to measure the elimination of the tracers over a specific time frame (43). The difference in the rate at which the tracers are eliminated from body water allows for the calculation of carbon dioxide production, a product of energy metabolism that is used to compute TDEE. The added tracers also can be used to calculate the individual's total body water. Based on total body water, fat-free mass and fat mass can be calculated. These equations are typically applied to DLW-derived total body water to determine body fat%, an age-related adjustment for chemical maturation of fat-free mass.

While DLW is an objective reference measure of TDEE and valid for body fat%, it is not practical for clinical use due to its high cost, specialized equipment and expertise that is required (43). See Table 5 for DLW variables and standard calculations.

**Table 5:** Doubly labeled water variables, calculations, and energy expenditure.

Variables	Calculations and operational definitions
Fat-free mass (FFM)	FFM (kg) = total body water (kg)/Hydration factor of FFM based on child's age & sex (44) FFM is all body components except fat tissue (45). FFM contains 73.2% water in healthy adults, but in children the hydration of FFM is higher (46) and is based on age and sex and is listed in (47).
Fat%	Fat% = 100—[Weight-total body water/Hydration Factor of FFM based on child's age and sex (44)]/weight. The percentage of body weight that is fat. The equation uses the hydration factor of FFM for children (46)
Total body water (TBW)	TBW = [(N_O_/1.007) + (N_D_/1.043)]/2, where N_i_ = [(W × A/a) × (ΔDD/ΔBW)]/(1,000)—[cumulative fluid intake (kg)] (43) The water content of the body at birth is 70%–75% but decreases into adulthood. Water (intra and extracellular) is contained exclusively within the fat-free mass (43). The TBW was calculated from the dilution space of oxygen-18 (N_O_) and deuterium (N_D_) in the body water pool adjusted by the non-aqueous exchange values 1.007 and 1.043 for each isotope, respectively. In the calculation, N_i_ is the dilution space for each isotope, “W” is the amount of water used to prepare the diluted dose, and “a” is the amount of DLW used in this dilution. “A” is the dose of DLW consumed by the participant, ΔDD is the ^2^H or ^18^O enrichment measured in the diluted dose, and “ΔBW” is the plateau enrichment measured in body water on Day 0 (43).
Total energy expenditure (TEE) & TDEE	TEE (kcal/day) = *r*CO_2_ × (1.106 + 3.94/RQ) (48) TEE is the total energy used by our body to maintain life (49) including basal metabolic rate, thermic effect of food, and physical activity (and growth in children) (19). In the TEE calculation, the rate of CO_2_ production (*r*CO_2_) is based on the body's elimination of isotopes (*r*CO_2_ = 0.4554 × TBW × [(1.007 × k_O_)—(1.043 × k_D_)] × 22.26), on which the 22.26 is the gas constant for carbon dioxide in L/mol; and RQ = respiratory quotient estimated from typical diet (48). In our study, TEE is measured over 7 days and TDEE is the 1-day average.

#### Weight-related behaviors

Sleep and activity data are collected by an accelerometer and activity tracker that are worn day and evening but removed during water activities. See Table 6 for physical activity assessment devices.

**Table 6:** Physical activity monitors.

Monitor	Operational descriptions
ActiGraph GT3X-BT Accelerometer	The ActiGraph Accelerometer GT3X-BT is worn on the waist Days 0–7 during awake and sleep hours and removed during water activities (e.g., swimming, bathing) to measure activity and sleep. Parents/LAR will record the time when the child goes to bed and wakes (estimated) using a daily log sheet. Raw data at 30 Hz will be collected and converted to counts for analysis. During Day 0, participants will be guided by the study team to complete a ramped protocol based on the individual's self-reported perceived exertion at baseline, light, moderate and vigorous intensity levels aided by a validated pictorial perceived exertion chart (42).
Fitbit Inspire 2 Activity Tracker	The Inspire 2 Fitbit is worn on the non-dominant wrist on Days 0–7 to provide a second-level heart rate measurement and a second objective measure of sleep and sleep quality (e.g., total sleep time, sleep efficiency, wake after sleep onset, sleep onset, and sleep offset).

#### Instruments

Parent/LAR complete all instruments with child assistance as able. All instruments are available in Spanish and English. See Table 7 for a detailed description of the instruments.

**Table 7:** Instruments.

Variable	Measure
Pubertal status	Parent report based on a pictorial and written definition of Tanner stages 1–5 (development of external genitalia for males and breast development for females). Tanner stage 1 = pre-pubertal and stages 2–5 = pubertal. The purpose of this study is not the specificity of the stage, but to discern pre-pubertal vs. pubertal. Parent report of youth was valid when compared to healthcare provider assessment [*r* = .75 to *r* = .87 (*p* ≤ .001, *k* = .13–.55)] (50).
Nutrition	Block Food Screeners for ages 2-17-2007 (51). This 41-item screening instrument assesses intake by food group by amount [3-point scale] and frequency [6—point scale] consumed in the last 7 days. When compared to 24-h dietary recalls, they were correlated between 0.526 for vegetables to 0.878 for potatoes. Bland-Altman plots indicated no systematic difference between the two based on food groups (52). Approximately 10–12 min to complete (51).
Function	PEDI-CAT (53). The Pediatric Evaluation of Disability Inventory Computer Adaptive Test measures abilities in ages 2–21 in four functional domains: daily activities, mobility, social/cognitive, and a responsibility domain. Uses Item Response Theory statistical model to estimate the ability from a minimal number of most relevant items. Positive reliability (ICC = 0.96–0.99) and discriminant validity between children with and without disabilities (*p* < .0001). Mean completion time of 12.66 min (SD = 4.47) (54).
Physical and sedentary activity	Block Kids Physical Activity Screener for ages 8-17-2003 (51). This 9-item tool assesses the frequency (6-point scale) and duration (4-point scale) of physical and sedentary activity in the last 7 days. When used to estimate kcals based on weight, age, intensity, frequency, and duration in 48 children against an accelerometer, partial correlations (controlling for age and weight) were significant (*r *= 0.56, *p* < .0001) (55). *Without a validated measure of physical activity for youth with disabilities, this instrument will provide additional context surrounding activity. Accelerometers will provide an objective measure of activity. Approximately 5 min to complete (51).
Child sleep habits questionnaire (CSHQ)	CSHQ (56) will be used with a sleep log and yields eight subscales, six of which are of interest in the current study: bedtime resistance, sleep onset delay, sleep duration, sleep anxiety, night awakenings, and daytime sleepiness. The retrospective questionnaire comprises 45 items; each is scored *usually* (1), *sometimes* (2), or *rarely* (3), and the parent indicates whether the sleep habit *is a problem (yes/no)*. The instrument has good internal consistency, reliability, and validity (57) and has been used successfully in children with developmental disabilities (58). We will calculate a global sleep problems score, and secondarily assess usual sleep latency and the number of awakenings. Approximately 10 min to complete (56).
Demographic form	Youth: age (month/year), ethnicity, sex, level of education, participation in an Individual Education Plan, and concurrent diagnoses. Parent/LAR: age, sex, marital status, relation to the child, family income, education, insurance, ethnicity, ZIP Code of residence, and work status. Approximately 5–10 min to complete.
Autism spectrum rating scale - ASRS (short form)	The ASRS (59) is a standardized, norm-referenced 15-item assessment that is available in English and Spanish and is a parent report measure designed to assess behaviors associated with autism spectrum disorder in children ages 2–18 years. The form includes a scale to assess behavioral rigidity, which we will use for the proposed study. Test-retest reliability ranges from .78 to .92 for the Total score and from .70 to .92 for the subscales. Internal consistency is high, ranging from .77 to .97; validity is also high. On average, the scales accurately predict group membership, with a mean overall correct classification rate of 92.1% (59). The ASRS subscale, behavioral rigidity, will be used to interpret patterns and quality of dietary intake. Approximately 5 min to complete.
24-hour dietary recall	A total of three (2 weekdays and 1 weekend day) multiple-pass 24-hour dietary recalls are conducted with the participant and parent/LAR during the study protocol. Days following an atypical event will be avoided. Recalls will be conducted via the virtual platform for the previous day's intake and adjunct supplies are provided to families (measuring spoons, cups, and a pictorial sheet on portions) to assist them in their accuracy of describing portion sizes. Estimated time per recall 30 min or less.
Parent-proxy peer relations	A validated PROMIS questionnaire that includes 7 questions related to the child's peer relationships answered by the parent/LAR with a five-point scale of never to almost always.

#### Retention

To ensure retention, we employ multiple strategies. Our initial strategy is to be clear and transparent as to what participation in the study involves. This allows the family to make an informed decision and be less likely to be surprised by the protocol which could lead to them to withdraw from the study. We also try to remain consistent with the study team member who is interacting with the family to support the building of a relationship and comfort with the study.

The next strategy is to support the family through the study. We provide information to guide the family verbally and in print with easy-to-follow instructions. We are flexible and available for their questions. Examples of being flexible include offering data collection visits on weekday and weekend mornings through early evening hours and accommodating the different time zones. We proactively consider potential concerns the family may have and provide reminders throughout the study. Our team is committed to reducing the study burden and supporting the completion of the protocol.

To accommodate unforeseen scheduling difficulties, we expect and accommodate appointment changes to the best of our ability. During the initial visit, we set up the additional two 24-hour dietary recalls (2 weekdays and 1 weekend day) per the family's schedule.

To support the families' participation in the study, each family is offered a $250 gift card for completing the study protocol. The gift card is not sent to the family until the Day 7 sample collections and supplies are returned and received by the study's coordinating center. The process and requirements for receipt of the gift card are discussed during the consenting process and reiterated during the Day 0 visit, so the family is aware of what to expect.

In summary, retaining study participants to complete the study protocol is enhanced by replicating strategies successfully used in our pilot study and current R01, including (1) the provision for a comprehensive explanation of what the study entails (aided by the pictorial study manual) so the family can make an informed choice to participate, (2) the provision of flexible scheduling options, (3) confirmation of understanding of the study protocol during the consenting process, (4) clear instructions provided verbally and in print, and (5) provision of all materials, clearly marked, that are needed for the collection and the return of the Day 7 urine samples, activity tracker and accelerometer with mailing supplies provided, and (6) touchpoints or interactions with the family during the study protocol to increase opportunities for questions, the reiteration of directions, and building of relationships with the family. To assist the family with the Day 7 urine collection, we call and/or text them on Day 5 and/or 6 to remind them of the final urine collection, review instructions, and answer questions.

While we do not anticipate challenges with retention based on the above strategies and our previous success, if we begin to have problems with retention, we will ask the families if they were willing to share what the challenges are and what led to their decision to not complete the study. Dealing with challenges early in the process allows us to make changes if able with our protocol and IRB amendments.

### Statistical data analysis

Descriptive statistics, including mean, standard deviation, median, and range will be calculated for each variable/measurement. Descriptive analysis will be conducted to assess for missingness, describe sample characteristics, and estimate the reliability of scores for all instruments. For body mass index (kg/m^2^), the average standing height measured will be used.

#### Missingness analysis

Univariate or regression analysis in both Aim 1 and Aim 2 will be performed using multiple imputation under the missing-at-random (MAR) assumption. Sensitivity analysis will be performed to check for potential violation of MAR assumption (60). Simulation under the MAR assumption will be performed to quantify the efficiency loss defined as 1−E [*Ŷ* (X*)−Y]^2^/E[*Ŷ* (X)−Y]^2^ where X* represent the observed data and the X is the data with missingness.

Analysis Aim 1, Bland-Altman plot analysis and concordance correlation will be used to evaluate the agreement between DLW body fat% and body mass index. We will predict TDEE by estimating basal metabolic rate and physical activity level and using these as our primary predictors. Several basal metabolic rate equations from cross-sectional studies with good model performance (i.e., *R*^2^ 0.7–0.8) have been reported^85^ which mainly used age, fat-free mass, and fat mass weight as key predictors. Thus, we will use those as our base model predictors and further consider height, race, sex, and pubertal status as potential predictors. Stepwise linear regression procedures in conjunction with Akaike information criterion (AIC) will be used for model selection and to develop prediction equations. Potential interactions between age/race and other variables will be examined. The resulting predicted TDEE is a weighted sum of a subset of potential predictors, where the weights are the parameter estimates associated with each predictor in the regression model. Predictive accuracy will be further evaluated by mean square error through a 10-fold cross-validation to test the predictive accuracy between the predicted and DLW TDEE.

Analysis Aim 2, The Block Food and Physical Activity Screeners, will be analyzed through Nutrition Quest, the child's function is scored through Pedi-Cat and the Autism Rating Scale is hand scored. The heart rate and sleep data obtained with the Fitbit activity tracker will be extracted and exported by Fitabase (Small Steps Labs LLC, San Diego, CA, USA.), a comprehensive data management platform. Descriptive analysis and univariate test (i.e., ANOVA or Chi-square test) on frequency and duration of physical activities, sleep, autism rating, sedentary activity, and youth's dietary intake by food groups and servings will be presented. Overall pattern by sex will be analyzed by principal component analysis or multiple correspondence analysis (61). Raw accelerometer signals at a sampling frequency of 30 Hz will be collected. Each data file will be manually cleaned to extract data collected and assessed for non-wear time using validated algorithms with ActiLife software (62). We plan to use the physical activity intensity thresholds based on counts per minute (CPM) that were validated in typically developing youth (63, 64); sedentary (<100 CPM), light (101–2,295 CPM), moderate (2,296–4,011 CPM), and vigorous intensity (>4,011 CPM). The CPM will be obtained based on a 10-second epoch to capture intermittent movement. We will also perform a 20-minute ramped protocol and collect participant perceived exertion and heart rate during Day 0 when parents can provide assistance as instructed. The ramped protocol will assist us in further validating the CPM thresholds with our participants with DS at individual levels and allow us to make any adjustments if needed based on raw data collected. The Physical Activity Screener will complement the objective accelerometer data. The 24-hour dietary recalls will be analyzed using Nutrition Data Systems for Research (65) (a nutrient analyses software) to identify energy intakes at the macronutrient levels and general Health Eating Index scores to compare with other youth groups and the Dietary Guidelines for Americans 2020–2025 (66).

### Sex as a biological variable analysis

The proposed sample of 230 youth with DS will be stratified by sex with 115 males and 115 females being recruited. For Aim 1, sex as a biological variable will be used to describe the average TDEE and tested as a potential predictor in the equation described above (Statistical Data Analysis, Aim 1) to predict energy requirements for youth with DS. This is important as TDEE is expected to be different for males as compared to females. For Aim 2, sex as a biological variable will be used to describe and examine differences between the weight-related behaviors (dietary intake, sleep, and activity) by univariate analysis (i.e., ANOVA or Chi-Square test as appropriate) for the sample based on sex. This will inform if obesity risk factors are different based on sex and used in future interventions.

### Power analysis

The power calculation is based on the increased coefficient of determination (*R*^2^) from a baseline linear model to a multivariate model that regresses TDEE on potential predictors (i.e., height, race, age, sex, puberty status). Pilot studies from the literature suggest that the *R*^2^ for a regression model of TDEE measured by DLW on basal metabolic rate and physical activity level is in the range of 0.7–0.8. As we will use 10-fold cross-validation to construct our predictive models for two sex groups and assume that 10% of subjects will not complete all measures based on Pilot One data, a sample size of 93 (230/2*0.9*0.9) achieves 90% power to detect an increase of *R*^2^ ≥ 0.045 attributed to additional four independent variables using an *F*-test with a significance level of 0.05. The variables tested are adjusted for two independent variables (basal metabolic rate and physical activity level) in the baseline linear model with an *R*^2^ of 0.7.

## Discussion

Individuals with DS are recognized to be at higher risk for obesity, yet there is insufficient evidence related to energy expenditure and weight-related behaviors that are integral to the prevention and treatment of obesity. This is critical as individuals with DS are living longer and the added burden of obesity can affect the individual's ability to self-manage their health and transition to independence. Furthermore, obesity is linked to multiple medical conditions. This lack of evidence-based information places added stress on the family and limits the ability of the healthcare provider to provide anticipatory guidance.

The study protocol was designed with the goal of obtaining clinically relevant information on TDEE and weight-related behaviors in youth with DS while reducing the burden on the child and their family. Specific burden-reducing attributes of this study include flexibility in scheduling and collecting data within the family's residence via a video conferencing platform along with the provision of family-centric, easy-to-understand study-related materials. This supports the family's ability to make an informed decision to participate, increases accessibility, supports recruitment efforts, aids in the collection of accurate data, and decreases the study burden on the participant. Post Covid-19, families have increased awareness and comfort with using virtual platforms such as Zoom which can be a benefit to conducting research studies and reduce the need to enter medical research facilities if not needed.

An added benefit of conducting the visit within the family's residence includes the ability to have the family be in a comfortable environment during data collection. This assists the child with comfort during the study and with obtaining data. A specific example of this is during the 24-hour dietary food recall, the family can go to their kitchen and show the study team actual serving sizes and brand names of food. The extended study visit allows the building of a relationship with the family and provides the ability to answer questions as they come up. The study is also designed to ensure that the family does not need to leave their house for participation by having all study-related equipment and supplies shipped to the family and picked up from the family's residence. The successful completion of this study with the use of virtual platforms, family-centric study materials, and a focus on reducing family burden during study participation may provide evidence for alternative study designs in the future.

The study outcomes will shift current research and clinical practice paradigms by providing an evidence-based prediction equation to support healthcare providers to provide accurate anticipatory guidance for youth with DS and their caregivers/families. Our protocol can serve as a template for research into other developmental disabilities and the virtual methodology can provide a model that supports study participation and potentially can increase the diversity of the sample. The Aim 1 evidence-based prediction equation for TDEE will be immediately useful and translatable into clinical practice. Our findings will also provide a requisite foundation for future intervention research, and when compared to our ongoing R01 findings, will generate knowledge about whether intervention components need to be tailored to specific disabilities (e.g., different TDEE in DS vs. SB) or if intervention components can be more broadly tested across populations. The findings from Aim 2 are foundational as the origin of obesity is multifactorial and can be influenced by behavioral modifications. The Aim 2 outcomes will inform both providers and future research on current weight-related behaviors as obesity risk factors in youth with DS. Evidence-based information on weight-related behaviors (nutrition, activity, sleep) can be used independently [e.g., recommendations for dietary modifications or opportunities to modify activity once we have a better understanding of the daily activities (physical and sedentary) of youth with DS] and with the TDEE information obtained in Aim 1 to developing interventions both from a clinical and research perspective.

The lack of accurate information on energy expenditure and weight-related behaviors (nutrition, activity and sleep) in youth with DS and the inability to provide daily caloric recommendations significantly impedes the successful prevention and treatment of obesity for this vulnerable population. The findings from this study will provide a foundational understanding of weight-related behaviors (energy expenditure, activity, nutrition, and sleep) as obesity risk factors, currently not well understood for this vulnerable population. This information will optimize clinical appointments and support and enhance the anticipatory guidance provided by healthcare providers. This innovative study will advance the science of weight management in individuals with disabilities, address national research priorities and shift clinical practice paradigms.

## Ethics statement

The study protocol is approved by the Western Copernicus Group (WCG) Institutional Review Board (IRB) (#20214186).
