# Erratum: Energy expenditure and weight-related behaviors in youth with down syndrome: a protocol

**DOI:** 10.3389/fped.2023.1276199

**Published:** 2023-08-29

**Authors:** Frontiers Production Office

**Affiliations:** Frontiers Media SA, Lausanne, Switzerland

**Keywords:** down syndrome, trisomy 21 (Down syndrome), nutrition, physical activity, energy expenditure, obesity, doubly labeled water (DLW), wearable devices

An Erratum on Energy expenditure and weight-related behaviors in youth with down syndrome: a protocol by Polfuss M, Bandini LG, Ravelli MN, Huang Z, Moosreiner A, Schoeller DA, Huang C-C, Ding D, Berry C, Marston E, Hussain A, Shriver TC and Sawin KJ (2023). Front. Pediatr. 11:1151797. doi: 10.3389/fped.2023.1151797

Due to a production error, the funding grant number “R01HD096085” for the “Eunice Kennedy Shriver National Institute of Child Health & Human Development of the National Institutes of Health INCLUDE (INvestigation of Co-occurring conditions across the Lifespan to Understand Down syndromE) Project” was omitted.

The publisher apologizes for this mistake.

The original version of this article has been updated.

